# B-Hex, an ace up the sleeve for small pupil phacoemulsification

**DOI:** 10.22336/rjo.2022.13

**Published:** 2022

**Authors:** Rimsha Sarosh, Omar Rashid

**Affiliations:** * Perfect Vision Superspeciality Eye Hospital, Gulshan Nagar, Nowgam, Srinagar, India

**Keywords:** B-Hex, small pupil, phacoemulsification, pupil expansion

## Abstract

**Objective:** To analyze the utility and the nuances of implanting the B-HEX® Pupil Expander (Med Invent Devices, India) at various stages in small pupil phacoemulsification.

**Methods:** This prospective interventional case series was undertaken to assess the utility of B-Hex in small pupil phacoemulsification under topical anesthesia. Our series comprised of 50 cataract cases with small pupils of various etiologies, operated by a single surgeon at our private practice, beginning February 2018. Cataract cases with pharmacological dilation of < 4 mm underwent phacoemulsification with B-Hex implantation. It was employed at the outset or interim, whenever the need arose.

**Results:** The B-Hex ring is extremely handy and useful in small pupil phacoemulsification. The average age of our study cohort was 62 years. In 38 cases B-Hex was employed before making a capsulorhexis. Progressive pupillary constriction during nuclear disassembly warranted the utilization of the device in 9 cases, while the remaining 3 cases had a retained epi-nuclear plate or significant cortex and the ring was placed after nucleus removal. No significant complication was noted. Postoperatively, the pupil dimensions, notably the architecture, were maintained. No significant AC reaction was noted. Intraocular pressure was not high.

**Conclusion:** Our experience showed that B-Hex is secure and easy to use under topical anesthesia. The learning curve is shallow and the technique is precise. B-Hex is truly an ace up the sleeve.

## Introduction

Small pupil phacoemulsification is no easy task, perchance be daunting for experienced surgeons as well [**1**]. Visibility is of utmost importance in safe cataract surgery. Conceivably, the overall rate of intra and postoperative complications like iris sphincter tears, iridodialysis, anterior capsule tears, zonular dialysis, posterior capsule rent, retained lens material, chronic inflammation, cystoid macular edema, are all seen more frequently in cases with small pupils [**2**-**4**]. The association of small pupils and the dreaded intraoperative floppy iris syndrome (IFIS) is well known. A maximum dilation of less than 7 mm is a vulnerability [**5**,**6**]. 

A graded sequential approach to pupil dilation (like the Peregrine Eye and Laser Institute Small Pupil Algorithm) [**7**] has been prescribed and is usually practiced universally. In modern times, expansion rings are the most useful mechanical devices. Iris hooks are more cumbersome, tedious, and time-consuming [**8**,**9**]. With the availability of devices like the Malyugin ring, Canabrava ring, Assia pupil expander, B-Hex, I ring, and Iris speculum, among others, the threshold for using pupil augmentation rings has come down [**7**,**10**-**12**].

In the real world, however, most adept surgeons can handle a soft cataract with 4 mm plus-sized pupils. To equip the new age surgeon, a range of techniques and devices is available to step up the pupil. In practice, very often, the complexity of the case is determined by the patient profile. A premium IOL coupled with a hard nucleus and an undersized pupil can be quite challenging. Historically, the tally of patients opting for the new age IOLs is at an all-time high.With the introduction of the third generation Bhattacharjee ring or the B-Hex (Med Invent Devices, India), the small pupil game has been revolutionized for the third world surgeon. The uniplanar, thin (0.075 mm) hexagonal silhouette of the ring coupled with the injector-free delivery and removal is revolutionary [**13**,**14**]. Our proposal is to appraise the utility of the B-HEX® Pupil Expander in cataract surgery through small pupils. 

## Methods

This study was envisioned as a prospective noncomparative interventional study. Our series of 50 small pupil cataract cases were operated by one designated surgeon. The cases with maximum pharmacological dilation of < 4 mm were defined as small pupil cases. We included cases of alpha antagonist use and suspected IFIS, pseudoexfoliation, post uveitis, rigid and fibrotic pupils, chronic pilocarpine use, and senile miosis. Cataract morphology included were nuclear sclerosis (including grade 4), posterior subcapsular, and cortical cataract. All cases were operated under topical drop anesthesia. Cases with subluxation, post-traumatic cataracts, patients not able to cooperate for topical surgery were excluded. After detailed pre-operative examination and counseling and informed consent, phacoemulsification under topical proparacaine supplemented with intracameral preservative-free lidocaine was scheduled. Pre-operatively, 1% tropicamide and 5% phenylephrine were used to expand the pupil, pharmacologically. A standard 2.8 mm clear corneal incision was placed on the steep axis, straddled by two side-port incisions 90 degrees away. After staining the anterior lens capsule using trypan blue, the anterior chamber was filled with cohesive viscoelastic, which is known to aid viscomydriasis. In the cases with pupils < 3 mm, a bimanual stretch was undertaken using Kuglens hooks directed 180 degrees apart, directed at 3-9 o’clock, followed by the 12-6 o’clock axis. It is pivotal to reinject a dash of viscoelastic underneath the iris to create adequate space, pushing the lens away. The chamber should not be overfilled. This improves the safety profile and curbs the chances of anterior capsular trauma.

Next, viscoelastic was injected over the ring in the housing provided, which was then placed snugly with the incision. The ring was grasped at the median positioning hole on a flange with 23 G forceps and advanced inside the anterior chamber. The B-Hex ring was gently positioned on top of the iris. The 23G forceps was then directed through the right-side port and the opposite flange was grasped at the mid-positioning hole. The ring was lifted off the iris and withdrawn until it advanced 1 mm beyond the iris margin. With a sure stroke, the curved notch engaged the iris margin and B-Hex was wriggled into position. This was followed by a similar tucking of the flange crossways via the opposite side port. If needed, viscoelastic was supplemented at this stage. The last flange, positioned at the 6’o clock was then tucked behind the iris and a comfortable 5.5 mm pupil was acquired. Since the last flange is often deemed as the most difficult to tuck, it is worthwhile to proceed in this sequence. Caution should be taken to keep the device parallel to the iris at all times and avoid pronation of the hand, thereby inducing a tilt. This tilt might cause engagement of only one notch.

A continuous curvilinear capsulorhexis was then made. Horizontal chopping with a blunt ball tip chopper was the favored technique used universally. Cortical wash was performed through bimanual irrigation and aspiration cannulas. A single-piece hydrophobic acrylic IOL was then injected into the bag. The 23 G forceps were used to disengage a notch, the ring was then grasped and fished out of the main incision. Viscoelastic was removed thoroughly and an intracameral injection of antibiotics was given. The main incisions and sideports were hydrated.

Postoperatively, slit lamp assessment was performed one hour after surgery and repeated the next day. Topical steroids were prescribed starting 5 drops a day and tapered as usual. Routine follow-up at one, two-, and four-weeks post-surgery was undertaken.

## Results

In our series of 50 cases, the preoperative evaluation revealed pupils < 4 mm in 38 cases. We stationed the device before performing capsulorhexis. This ensured a safe cataract surgery. In 9 cases, the pupil was adequate at the beginning of the surgery and a 5 mm capsulorhexis was fashioned. En route, however, there was progressive miosis during nuclear disassembly. These cases were IFIS and had iris prolapse from the main and sideports. The anterior chamber and the capsular bag were filled with viscoelastic and B-Hex was used. Phaco machine parameters were adjusted. In 7 cases, further iris prolapse did not occur. In two cases, the iris was still out from the main port. In the remaining three cases of IFIS, the B-Hex ring was employed after cataract aspiration, wherein a significant amount of epi-nuclear material was present behind the miosed pupil and precluded the safe removal.

The average age of our study cohort was 62 years. Out of the 50 cases, 29 were men and 21 were women. The patient and ocular characteristics are condensed in **Table 1** and **2**.

**Table 1:** Etiology of small pupil (Ocular)

Ocular characteristics	Number of Eyes
Pseudoexfoliation	22(44%)
Intraoperative floppy iris syndrome	12(24%)
Uveitis	6(12%)
Benign senile miosis	6(12%)
Topical pilocarpine use	4(8%)

**Table 2:** Etiology of small pupil (Systemic)

Patient characteristics	Number of Patients
Diabetes	17(34%)
Alpha agonist use	13(26%)

The average pupil diameter before surgery was 4.2 +/- 1.1 mm after maximal pharmacological dilation. The average pupil diameter was 4.6 +/- 1.1 mm one-hour post-surgery and 2.8 +/- 1.1 mm the next day. The one-month post-operative visit did not reveal any case of persistent pupil distortion other than the two cases of rigid pupils that had sphincter tears, despite utmost care during bimanual stretching. No patient complained of glare. Noncontact tonometry did not reveal a pressure of more than 22 mmHg in any case, one day after surgery, and was 14 +/- 2 mmHg at the two weeks follow-up. 

## Discussion

Small pupil phacoemulsification tests the surgeon’s skill set. Modern-day cataract surgeons have an armamentarium of assistive pupil expansion devices at their disposal. Gone are the days when liberal sphincterotomies and key-hole iridectomy were inevitable for small pupils, as would the consequent glare and unhappiness. At an individual level, the high-volume cataract surgeons may prefer to proceed without an expander in cases in which they feel comfortable. However, it is prudent to use a pupil expansion strategy when the pupil is less than 4 mm. Pupil expansion devices may require the recruitment of additional skills, instruments, and potential costs. All these may come with additional risks like sphincter tears, hyphaema, anterior capsular tears, and persistent pupil distortion among others. A minimal push-pull technique with two iris hooks has also been described [**15**]. Essentially, each surgeon decides what suits them best, aiming solely at a safe surgery.

B-Hex has a fine uniplanar design and is very flexible. Our case series comprised 50 poorly dilated pupils with diverse etiologies like pseudoexfoliation, posterior synechiae associated with uveitis, angle-closure, and benign senile miosis.

In cases with pseudoexfoliation, the pupils are rigid and dilate poorly. These eyes are considered difficult as a consequence of the poor visibility and zonular instability that is quite common in them. A review of literature shows that pseudoexfoliation is largely considered to be an absolute contraindication for multifocal lenses and a relative contraindication for the toric lenses considering the prospect of late decentration and tilt, which would compromise the visual quality tremendously in multifocal and toric lenses [**16**].

The success of cataract surgery in cases with uveitis is dependent on pre- and post-operative control of inflammation. Care was taken to adhere to a set protocol and peri-operative steroid cover was employed for these patients. These cases do great with the B-Hex ring. The incredibly thin and flexible profile of the ring makes it ideal for use in them. In eyes with posterior synechiae, synechiolysis was done and the pupil stretched before the implantation of the ring. Even in these chronically inflamed irides, the B-Hex ring does not cause much additional tearing and is convenient to place and remove. Minimal bleeding occurred in two cases after stretching of the pupil. Postoperatively, inflammation and IOP were commensurate with expectation. Long-acting beta-blocker preparation was used topically wherever necessary. Since eyes with uveitis are always at enhanced risk of reactivation and long-standing inflammation, it was deemed prudent to steer clear of premium lenses [**16**].

Eyes with angle closure, on chronic pilocarpine therapy, also present with problems in expanding the pupil for cataract surgery.

**Fig. 1:**
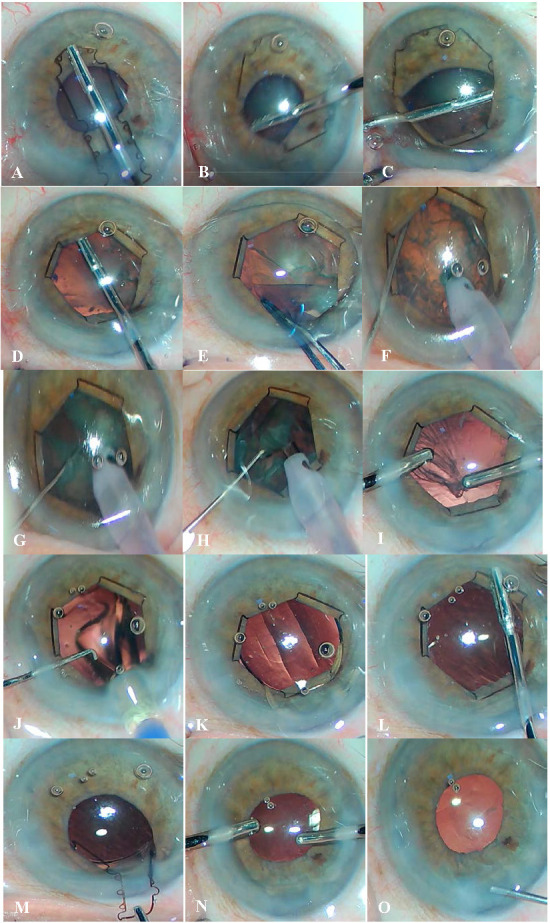
Case 1: Implantation of the B-Hex ring in a case with Pseudoexfoliation. **A.** Insertion of the B-Hex ring into the anterior chamber; **B.** Engagement of the first flange through the right side-port; **C.** Tucking of the second flange through the left side-port; **D.** Tucking of the third flange; **E.** Capsulorhexis is fashioned with the Utrata forceps; **F.** Horizontal chopping technique; **G.** The first chop; **H.** Emulsification of the nucleus; **I.** Bimanual technique of cortex removal; **J.** Injection of the foldable single-piece acrylic Intra-ocular lens (IOL); **K.** IOL in the bag; **L.** Disengaging a flange with notches; **M.** The B-Hex ring is pulled out from the main incision; **N.** Viscoelastic removal; **O.** Incision hydration

**Fig. 2:**
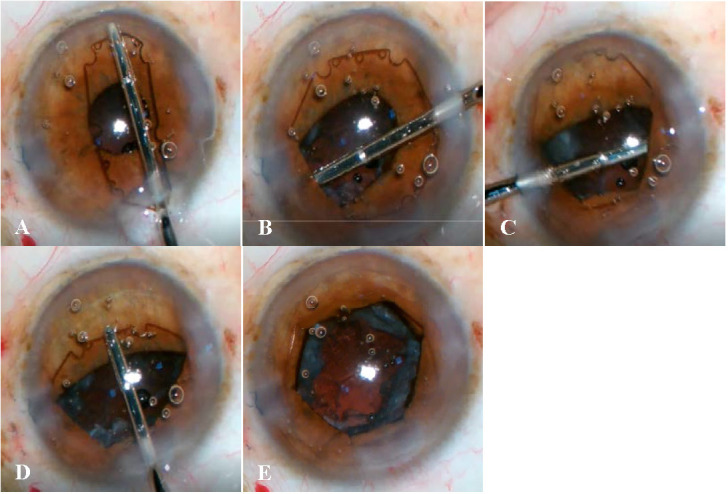
Case 2: A case of IFIS, the B-Hex ring was employed after phacoemulsification to ensure the safe removal of the epi-nucleus and cortex is retained behind the miosed pupil. **A.** The B-Hex ring is placed on the Iris; **B.** The positioning hole is grasped and the first flange tucked safely; **C.** The second flange is wriggled into position; **D.** The third flange engages the iris at the 6 o’clock position; **E.** B-Hex in position

B-Hex ring has been immensely useful in our practice for small pupil phacoemulsification, by way of hassle-free insertion and removal. Surprisingly, there is a dearth of published research regarding its use, reflecting in turn that it is yet to attain routine acceptance. This study intended to provide tips and safety data, to encourage surgeons to try the B-Hex ring. Our aim was to share our experience with this ring and assess a multitude of aspects concerning its use (**Fig. 1 A-O**, **Fig. 2 A-E**).

*Ease of insertion*

B-Hex ring is easy to employ and place in position. The time needed to place the ring was as low as 44 seconds and was never more than 90 seconds, whereas the removal takes 10 seconds. This concurs favorably with the study of Nderitu et al., wherein consultant ophthalmic surgeons required an additional time of 14 minutes for iris hooks as against 4 minutes for pupil expansion rings [**9**].

While holding the flange, grasping the central hole should be ensured by employing the tip of the forceps, for a better grip. The focus should be on the two notches at the ends of the flange, with side-to-side wriggly movements, making sure that both notches engage the iris margin. Counter traction can be provided by a second instrument lodged in the opposite side-port. If one or both notches slip behind the iris, it is safest to halt and disengage the ring rather than proceed to tuck the rest. The slipped notches can tear the anterior capsule. The flanges and notches should be tucked carefully and the examination of the anterior capsule in this area should be assured.

In practice, the ease of tucking decreases from the foremost to the third flange.

We propose a simple method of insertion. After placing the B-Hex ring over the iris across the main incision, the instrument was withdrawn. We went through the right-side port first and tucked the left flange with the right hand, withdrew again and went through the left side port to tuck the right flange. Viscoelastic was introduced as per need and the flange tucked at 6 o’clock. In pupils < 3 mm, it is important to stretch out the pupil before using the ring. If this is omitted, the device can buckle under pressure during the introduction of the third flange.

We introduced the ring in 12 cases of IFIS after progressive pupil constriction hampered visualization. B-Hex was implanted at various stages of nucleus removal in 9 cases and dealt with a retained epinuclear plate in 3 cases. Liberal viscoelastic injection beneath the iris plane pushed the remaining lens matter deep and improved the safety profile for implanting the ring at that stage. A second instrument could be pragmatic to lift the iris and improve visualization.

*Effectiveness of pupil augmentation*

The ring imparts an effective 5.5 pupil size, which is quite comfortable for a competent surgeon. Boris Malyugin defines the threshold for performing phacoemulsification for experienced surgeons in the range of 4.5-5 mm and recommends a pupil augmentation strategy for a smaller-sized pupil [**10**]. An area of 5.5 mm diameter provides enough space for performing capsulorhexis and nucleus disassembly. Technically, hexagon shape is advantageous with no wasted space and an excellent safety profile [**13**]. 

*The maneuverability of the phaco handpiece and instruments*

The thin profile and petite form of the B-Hex ring ensure good instrument mobility inside the eye and do not hamper their maneuverability. It can be used efficiently in eyes with shallow anterior chambers. Demonstrably, no impedance to the movement of the phaco handpiece, second instrument, irrigation/aspiration cannulas, and the IOL injector is encountered. We used a horizontal chopping technique and never decoupled the ring from the iris margin after striking with the chopper or nuclear pieces.

*Fluidics and anterior chamber stability*

The B-Hex ring provides excellent chamber stability and is invaluable on account of its ease of passage through small (1 mm) incisions. It provides a stable and dependable pupil expansion in the eyes with IFIS. B-Hex is very useful in mechanically supporting the elastic iris in these cases. It can safely be enlisted at any step of the surgery.

*Ease of removal*

The technique of revocation of the B-Hex is truly the most exciting feature. A notch is simply disengaged and the ring is decoupled from the iris before fishing out of the eye. The trailing notches let go of the iris margin atraumatically and do not pull it along. Alternatively, two flanges can be disengaged before the removal. Due to its flexible nature, the ring does not hitch or snag the incision [**13**]. It can even be maneuvered out the side port, although it is preferred to be removed via the main incision. The B-Hex ring did not break or buckle during introduction or revocation.

*Post-operative pupil architecture*

The mean pupil size achieved pharmacologically was 4.2 mm. Postoperatively, we noted round pupils with no significant distortion in 48 pupils. In the remaining cases, the iris showed sphincter tears. These cases had rigid and fibrotic pupils. No patient complained of glare. 

*The defining practical aspect-Cost*

It is a reality that a safe surgery is priceless and we, as surgeons, do not hesitate before using assistive devices for patient safety and surgical ease. The Malyugin Ring is magnificent and is truly pioneering work, nonetheless, it is also almost 4.5 times more expensive than the B-Hex ring in the Indian market.

The B-Hex ring is novel and exciting. It has not been extensively studied yet. A recent clinical trial by Salviat F, Febbraro JL, Zuber K et al. concentrated on the first-generation Bhattacharjee ring. The trial comprised of 21 cases wherein the pupil was < 6 mm and found the ring to be valuable for stable pupil expansion. They faced two intraoperative complications. One of the rings broke before implantation and another ring was deemed unstable and was explanted before the surgery ended [**17**]. Our impression of the B-Hex ring was favorable in this regard. None of the rings employed in this study or the ones before and since the conclusion of this study, broke or loosened. The third generation hexagonal B-Hex ring is more user-friendly and technologically advanced than the first-generation ring.

In their case series of 4 cases of phacovitrectomy, Chakraborty et al. found the B-Hex ring to be stable, despite the extensive turbulations in the eye encountered in posterior segment surgeries.

As in our series, they did not encounter any instance of spontaneous disengagement of the pupil margin from the ring. They concluded the ring to be very flexible, easy to place and remove [**18**].

Pupil expansion devices are a blessing for cataract surgeons. A recent comparative study amongst 6 popular pupil expansion strategies i.e., bimanual pupil stretch (group I), pupil radial cut open (group II), iris-retractor hooks (group III), OASIS iris expander (group IV), and Malyguin-ring (group V), B-HEX Pupil Expander (group VI) with 120 eyes, distributed evenly among the 6 groups (20 eyes each), showed no statistical difference between the groups on the parameters of postoperative visual acuity and intraocular pressure. Corneal endothelial density was also studied and they inferred those cases with iris retractors and Malyugin ring had the best endothelial counts.

The authors encountered instances of the B-HEX pupil expander falling off from the pupil and attributed it to the thin profile [**19**]. We did not encounter this complication in our 50 cases.

## Conclusion

Performing cataract surgery in eyes with small pupils is challenging as poor visibility compounds the difficulty level. Nowadays, surgeons have a myriad of pupil augmentation devices to choose from to ensure a safe surgery. All the available devices are safe to use and in the past few years, the threshold of using one has come down. The B-Hex ring is very fine, yet resilient, provides effective pupil expansion, and is easy to insert and remove. We hope that we have been able to provide evidence supporting the safety and ease of using the B-Hex ring.

What was known: B-HEX is a petite, uniplanar pupil augmentation device that is injector independent and can be introduced via 1 mm incisions.

What this paper adds: A safe insertion technique with tips on engagement and disengagement with the largest series in literature until present.

**Conflict of Interest statement**

None of the authors has any conflict of interest to disclose.

**Informed Consent and Human and Animal Rights statement**

Informed consent has been obtained from all individuals included in this study.

**Authorization for the use of human subjects**

Ethical approval: The research related to human use complies with all the relevant national regulations, institutional policies, is in accordance with the tenets of the Helsinki Declaration, and has been approved by the review board of Perfect Vision Superspeciality Eye Hospital, Gulshan Nagar, Nowgam, Srinagar, India.

**Acknowledgements**

None.

**Sources of Funding**

No funding or support was received for this study.

**Disclosures**

No financial disclosures.
